# Determining minimal clinically important differences in the Hammersmith Functional Motor Scale Expanded for untreated spinal muscular atrophy patients: An international study

**DOI:** 10.1111/ene.16309

**Published:** 2024-04-24

**Authors:** Giorgia Coratti, Francesca Bovis, Maria Carmela Pera, Mariacristina Scoto, Jacqueline Montes, Amy Pasternak, Anna Mayhew, Robert Muni‐Lofra, Tina Duong, Annemarie Rohwer, Sally Dunaway Young, Matthew Civitello, Francesca Salmin, Irene Mizzoni, Simone Morando, Marika Pane, Emilio Albamonte, Adele D'Amico, Noemi Brolatti, Maria Sframeli, Chiara Marini‐Bettolo, Valeria Ada Sansone, Claudio Bruno, Sonia Messina, Enrico Bertini, Giovanni Baranello, John Day, Basil T. Darras, Darryl C. De Vivo, Michio Hirano, Francesco Muntoni, Richard Finkel, Eugenio Mercuri, the ISMAC group, Laura Antonaci, Roberto De Sanctis, Sara Carnicella, Nicola Forcina, Giulia Norcia, Giulia Stanca, Lavinia Fanelli, Giacomo De Luca, Adelina Carlesi, Giulia Colia, Chiara Bravetti, Diletta Rossi, Rafael Rodriguez‐Torres

**Affiliations:** ^1^ Pediatric Neurology Università Cattolica del Sacro Cuore Rome Italy; ^2^ Centro Clinico Nemo Fondazione Policlinico Universitario Agostino Gemelli IRCCS Rome Italy; ^3^ Department of Health Sciences (DISSAL) University of Genoa Genoa Italy; ^4^ Dubowitz Neuromuscular Centre UCL Great Ormond Street Institute of Child Health & MRC Centre for Neuromuscular Diseases London UK; ^5^ Columbia University Irving Medical Center New York New York USA; ^6^ Boston Children's Hospital Harvard Medical School Boston Massachusetts USA; ^7^ John Walton Muscular Dystrophy Research Centre Newcastle University and Newcastle Hospitals NHS Foundation Trust Newcastle Upon Tyne UK; ^8^ Department of Neurology TD Stanford University Palo Alto California USA; ^9^ St. Jude Children's Research Hospital Memphis Tennessee USA; ^10^ NEMO Clinical Center Milan Italy; ^11^ Unit of Neuromuscular and Neurodegenerative Disorders Bambino Gesù Children's Hospital IRCCS Rome Italy; ^12^ Center of Myology and Neurodegenerative Disorders IRCCS Istituto Giannina Gaslini Genoa Italy; ^13^ Department of Clinical and Experimental Medicine University of Messina Messina Italy; ^14^ Department of Biomedical Sciences for Health University of Milan Milan Italy; ^15^ National Institute for Health Research Great Ormond Street Hospital Biomedical Research Centre London UK

**Keywords:** Hammersmith Functional Motor Scale Expanded, minimal clinically important differences, minimal detectable change, spinal muscular atrophy

## Abstract

**Background and purpose:**

Spinal muscular atrophy (SMA) is a rare and progressive neuromuscular disorder with varying severity levels. The aim of the study was to calculate minimal clinically important difference (MCID), minimal detectable change (MDC), and values for the Hammersmith Functional Motor Scale Expanded (HFMSE) in an untreated international SMA cohort.

**Methods:**

The study employed two distinct methods. MDC was calculated using distribution‐based approaches to consider standard error of measurement and effect size change in a population of 321 patients (176 SMA II and 145 SMA III), allowing for stratification based on age and function. MCID was assessed using anchor‐based methods (receiver operating characteristic [ROC] curve analysis and standard error) on 76 patients (52 SMA II and 24 SMA III) for whom the 12‐month HFMSE could be anchored to a caregiver‐reported clinical perception questionnaire.

**Results:**

With both approaches, SMA type II and type III patients had different profiles. The MCID, using ROC analysis, identified optimal cutoff points of −2 for type II and −4 for type III patients, whereas using the standard error we found the optimal cutoff points to be 1.5 for improvement and −3.2 for deterioration. Furthermore, distribution‐based methods uncovered varying values across age and functional status subgroups within each SMA type.

**Conclusions:**

These results emphasize that the interpretation of a single MCID or MDC value obtained in large cohorts with different functional status needs to be made with caution, especially when these may be used to assess possible responses to new therapies.

## INTRODUCTION

Spinal muscular atrophy (SMA) is a rare, progressive neuromuscular disorder that affects the motor neurons of the spinal cord, resulting in muscle weakness and atrophy [1]. In individuals with SMA, the survival motor neuron 1 (*SMN1*) gene is either missing or nonfunctional, leading to a shortage of the SMN protein and the eventual death of motor neurons [2]. SMA is categorized into different types based on the age at onset, clinical severity, and motor milestone achievements [3, 4]. Type I SMA, also known as Werdnig–Hoffmann disease, is the most severe form, manifesting in early infancy with progressive paralysis and leading to a significantly shortened life expectancy without therapeutic intervention. Type II SMA emerges in early childhood, causing moderate to severe motor impairment, whereas type III SMA typically presents after 18 months of age and leads to relatively milder motor dysfunction. In addition to the historical classification of SMA, functional classification of SMA is crucial for understanding the disease progression and designing appropriate interventions. SMA is typically categorized into three main functional classes based on the individual's ability to sit and walk independently. Nonsitter SMA individuals are those who are unable to sit independently and may require support for trunk control. Sitter SMA individuals can sit independently but are unable to walk. Finally, walker SMA individuals demonstrate the ability to walk independently for at least 10 m.

Over the past few years, there have been significant advancements in the treatment of SMA, including the development of disease‐modifying therapies [5]. Whereas in type I, the severe form of SMA historically associated with rapid progression and death, efficacy can be measured by increased survival and acquired or lost developmental milestones, in the more slowly progressive type II and III SMA possible changes were assessed using structured functional scales, such as the Hammersmith Functional Motor Scale Expanded (HFMSE), a scale specifically designed to assess functional changes in SMA that is commonly used in clinical trials, natural history studies, and often in clinical practice [6, 7, 8, 9, 10, 11]. The HFMSE, together with other functional scales, has been able to detect differences between treated and placebo arms in clinical trials and between treated patients and natural history‐matched cohorts in published real world data [12, 13]. As the magnitude of changes is variable, often in relation to the age and baseline values at the time when treatment is started [14, 15, 16, 17], there has been increasing pressure to assess the meaningfulness of the changes for the patients and their carers, using interviews and patient‐reported measures, and to use appropriate tools to establish minimal clinically important difference (MCID) values [18, 19, 20].

MCID is a statistical concept that is essential in determining the clinical effectiveness of treatments and interventions. It refers to the smallest difference in a score or measure that patients perceive as clinically meaningful or significant. This can be calculated using several methods, including Delphi methods, distribution‐based methods, and anchor‐based methods. Distribution‐based methods are based on statistical psychometric properties of the scale and provide a minimal detectable change (MDC), whereas MCID anchors minimal change on a clinical value of significance to the patient, clinician, or other stakeholders. MDC and MCID are both measures of clinical significance, but they have different meanings and applications [21, 22].

MDC refers to the smallest change in a measurement that can be detected with a certain level of confidence, usually 95%, based on the measurement error or variability of the instrument. It helps determine whether an observed change in a patient's clinical status is real or simply due to measurement error. In other words, MDC represents the threshold of detectability of an instrument, and it is used to evaluate the reliability and sensitivity of an outcome measure. On the other hand, MCID, calculated with anchor‐based methods, is defined as the smallest difference in score in the domain of interest that patients perceive as beneficial and would mandate, in the absence of troublesome side effects and excessive cost, a change in the patient's management or function [23]. MCID reflects the magnitude of change that is needed for a patient to perceive a difference in their health status. Anchor‐based methods use external criteria, such as patient‐reported outcomes or clinical global impressions [24, 25].

Recently, the US Food and Drug Administration (FDA) guidance document suggested that the MCID should be established using a combination of anchor‐based and distribution‐based methods [26, 27]. The guidance document emphasizes that the MCID should be established based on the clinical context and the specific study population and that it may vary depending on the severity of the disease and the baseline level, encouraging a patient‐level analysis rather than a between‐group analysis.

Most of the natural history studies and performed or ongoing clinical trials have used the HFMSE to measure functional changes over time [12, 13, 14, 15, 16, 28, 29, 30, 31, 32, 33, 34, 35, 36]. So far, there is limited information on MCID when using HFMSE, with only one cross‐sectional study using distribution‐based methods to identify values in adults with SMA [18].

This study aims to calculate the MDC and MCID values for the HFMSE in a large international cohort of SMA type II and III patients of all ages, considering age‐ and function‐related differences in disease progression trajectories. Both anchor‐based and distribution‐based methods were employed for this purpose.

## METHODS

This study retrospectively analyzes data from untreated SMA II and III individuals, collected prospectively as part of an international multicenter registry, the iSMAR [37]. As part of the activities of the registry, the study was approved by the ethical committees (ethical/institutional review board) of all participating centers, including as national/state coordinators the Catholic University in Rome, the UCL Institute of Child Health & Great Ormond Street Hospital in London, Columbia University Medical Center in New York, Harvard Medical School in Boston, Newcastle University in Newcastle, Stanford University in Stanford, and the University of Central Florida College of Medicine in Orlando. In adherence to ethical standards, all participants or their guardians provided written informed consent, which was approved by the relevant institutional review boards.

As part of the clinical routine at all participating centers, all patients are regularly assessed using the HFMSE, a functional scale designed to evaluate motor function in individuals with SMA. The HFMSE is a widely recognized and validated instrument that allows health care professionals to monitor changes in motor abilities over time, assess disease progression, and track the effectiveness of interventions or treatments [9, 10, 12, 13, 15, 16, 28, 29, 30, 31, 32, 33, 34, 35, 36, 38]. Its consistent use across centers ensures standardized and reliable data collection, enabling comprehensive evaluations of patients' functional status and facilitating comparisons and collaborations in multicenter studies. The HFMSE consists of a series of 33 motor tasks that assess different aspects of motor performance, including lying and rolling, sitting, crawling and kneeling, standing, and walking. Each item is scored on a 3‐point scale: 0, the individual cannot perform the task; 1, the individual can partially perform the task; 2, the individual can fully perform the task. The maximum possible score on the HFMSE is 66, with higher scores indicating better motor function [10]. As per standards of care, HFMSE assessments are conducted at least every 6 months [6].

The study included patients diagnosed with type II or III SMA, confirmed by genetic and clinical tests, if they had at least two evaluations, with one being at least 12 months after the initial assessment. Patients were excluded if one of their evaluations was deemed unreliable due to temporary issues like pain, fractures, recent illness, and scoliosis surgery. We also excluded data from patients involved in interventional clinical trials.

For the distribution‐based MDC calculation, to maximize the number of participants, multiple 12‐month observation intervals were defined for each participant, where 12 months was defined as two visits that were at least 304.2 days (47.3 weeks) and no more than 425.8 days (64.7 weeks) apart [30, 39].

The population was divided into groups based on historical and functional classification, with no analysis conducted concerning *SMN2* copy number. Notably, 40% of the population had three *SMN2* copy numbers, and for another 38%, the number remained unknown. Consequently, meaningful comparisons were not feasible.

### Measuring minimal clinically important difference

As per FDA guidelines [27], in this study, MDC and MCID were both employed to assess the magnitude of the change in functional changes, which was evaluated using the HFMSE. The MDC calculated using distribution methods is based purely on measurement error and does not take into account factors such as patient characteristics or disease progression [40]. As such, it may not be an accurate representation of the MCID [27].

For the anchor‐based method, the HFMSE function was compared with a caregiver‐reported clinical perception questionnaire (CRCP) adapted from a previously published questionnaire [41]. This comparison was conducted over a 12‐month period. The CRCP collected information from caregivers regarding the patients' disease progression over the past year and their expectations for the near future. The first two questions in the CRCP assessed the caregiver's perception of the patient's overall function during the past year and their expectations for the next 2 years. To calculate the MCID, only the first question was used, which asked caregivers to indicate whether the person they cared for had remained stable, experienced deterioration, or shown improvement in abilities during the past year.

The MCID analysis was performed on a subgroup of subjects who had both HFMSE and CRCP data available over a 12‐month period. Only patients with concurrent 12‐month HFMSE and CRCP data were included in the MCID analysis.

To further examine the MCID values based on age and functional status, the MDC was also calculated. This involved investigating score changes in different age and SMA function categories using a sizable population of SMA patients.

### Statistical analysis

#### Anchor‐based method

The anchor‐based method was applied using the results of a questionnaire assessing carers' perception of the progression of the disease [24, 40, 41]. One of the questions of the questionnaire investigated whether, over the previous year, it was felt that there had been stability, improvement, or deterioration. Patients were therefore categorized into three groups based on their CRCP (decline, stability, improvement). Correlations between changes in HFMSE and CRCP anchor values from baseline to 12‐month evaluation were calculated using a nonparametric Spearman rank correlation coefficient to assess the level of confidence in the interpretation of results.

The ability of the Hammersmith score to distinguish patients who feel that they have improved, been stable, or have worsened from the previous year was assessed through two methods:

- Receiver operating characteristic (ROC) curve analysis (ANCH‐A). The MCID was identified as the point of the ROC curve at which sensitivity and specificity were maximized (maximum [sensibility + specificity − 1], Youden index). The area under the curve was calculated to measure the instrument responsiveness and can be interpreted as the probability of correctly identifying the improved/stable patients from the deteriorated patients.
- The HFMSE 1‐year mean change from baseline was calculated in the whole cohort and according to age, HFMSE values, and functional status (Non sitter, Sitter, Walker; ANCH‐B) using stratification criteria identified in recent studies [11, 42]. MCIDs (and 95% confidence intervals [CIs]) for the HFMSE were determined by subtracting the mean change from baseline in the HFMSE score of the “stable” group from the mean score of “better” (MCID of improvement) and “worse” (MCID of deterioration).

#### Distribution‐based method

Distribution‐based methods rely on the SD of the measurement instrument and the reliability of the measurements. The MDC is the minimum change that must be observed in the score of an instrument measuring a symptom to be considered greater than measurement error and within‐subject variability [43].

To calculate MDC using distribution methods, two main approaches were taken:

- Calculate the standard error of measurement (SEM), indicating the precision of the outcome measure. This has been estimated as SD of the score at baseline multiplied by $\sqrt{1-\text{reliability}}$, where *reliability* is the test–retest reliability value and corresponds to 0.959 [38]. Set the tolerance interval (TI) based on a desired level of confidence. The TI is expressed as a *z*‐score, representing the smallest score change that can be detected beyond measurement error within a TI. For instance, a *z*‐score of 1.96 corresponds to a 95% level of tolerance. Finally, multiply the SEM by the *z*‐score. For example, if the SEM is 3 and the desired TI is 95%, the MDC would be 5.88 (3 × 1.96).
- Effect size change (ESch). We calculated the MDC as 0.5*SDch [44], where SDch represents the SD of the difference between the evaluation at baseline and at 12 months.

To account for repeated measures associated with the same subjects, baseline mean score and the SD of the change were obtained using a generalized linear model.

Categorical variables were reported as *n* (%), and continuous variables were reported as mean (SD). SAS version 9.4 was used to conduct all statistical analyses.

## RESULTS

### Anchor‐based methods

HFMSE 1‐year change and concurrent CRCP questionnaire were available for 76 subjects: 52 SMA II patients and 24 SMA III patients. Table 1 summarizes the baseline characteristics of the population analyzed.

**TABLE 1:** Characteristics at baseline of patients enrolled, by SMA type (whole population, anchor‐based).

Characteristic	All, *n* = 76	SMA type II, *n* = 52	SMA type III, *n* = 24
Age, years, mean (SD)	10.00 (5.78)	9.75 (6.11)	10.53 (5.08)
Adults *n* (%)	7 (9.21)	5 (9.62)	2 (8.33)
Sex, *n* (%)			
Female	34 (44.74)	19 (36.54)	15 (62.50)
Male	42 (55.26)	33 (63.46)	9 (37.50)
*SMN2* copy number, *n* (%)			
2	5 (6.58)	5 (9.62)	0 (0.00)
3	44 (57.89)	35 (67.31)	9 (37.50)
4+	7 (9.21)	1 (1.92)	6 (25.00)
Unknown	20 (26.32)	11 (21.15)	9 (37.50)
SMA function, *n* (%)			
Non sitter	10 (13.16)	10 (19.23)	0 (0.00)
Sitter	47 (61.84)	42 (80.77)	5 (20.83)
Walker	19 (25.00)	0 (0.00)	19 (79.17)

Abbreviation: SMA, spinal muscular atrophy.

### Correlation between change in HFMSE score and CRCP

Table 2 shows caregiver perception in the cases for which annual HFMSE 12‐month changes were available. In the analyzed population, those who reported an improvement in their health status over the past year had an average increase of 1.17 (95% CI = −0.98 to 3.31) in the HFMSE score if they had SMA type II, and average increase of 1.67 (95% CI = −2.46 to 5.79) if they had SMA type III.

**TABLE 2:** Hammersmith score 1‐year change by SMA type and caregiver‐reported clinical perception.

	SMA type II		SMA type III	
	*n* (%)	Mean change (95% CI)	*n* (%)	Mean change (95% CI)
Improved	12 (23.08)	1.17 (−0.98 to 3.31)	6 (25.00)	1.67 (−2.46 to 5.79)
Stable	28 (53.84)	−0.29 (−1.08 to 0.51)	7 (29.17)	−0.71 (−3.90 to 2.48)
Deteriorated	12 (23.08)	−3.5 (−5.98 to −1.02)	11 (45.83)	−3.91 (−6.84 to −0.97)

Abbreviations: CI, confidence interval; SMA, spinal muscular atrophy.

Based on 52 observed annual changes in SMA II and 24 in SMA III, a statistically significant moderate correlation between change in HFMSE score and caregiver perception groups was found from baseline to month 12 (*r* = 0.48, *p* < 0.0001). In SMA III patients the correlation is moderate (*r* = 0.51; *p* = 0.011), whereas in SMA II patients it is modest (*r* = 0.44, *p* = 0.001).

### Estimates of MCID

**FIGURE 1:**
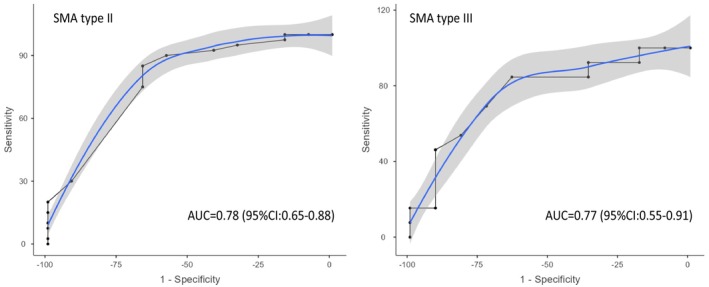
Receiver operating characteristic curves to discriminate improved/stable patients from the deteriorated patients for spinal muscular atrophy (SMA) type II and III. AUC, area under curve; CI, confidence interval.

Table 3 and Figure 1 present the MCID and the optimal cutoff points for SMA type II and SMA type III patients. The optimal cutoff points obtained by the ROC curve (ANCH‐A) that discriminates improved/stable patients from the deteriorated patients is −2 for type II patients and −4 for type III patients.

**TABLE 3:** Anchor‐based method (ANCH‐A and ANCH‐B) results.

	SMA type II	SMA type III
ANCH‐A		
	**>−2**	**>−4**
AUC (95% CI)	0.78 (0.65–0.88)	0.77 (0.55–0.91)
Sensitivity (95% CI)	0.85 (0.70–0.94)	0.85 (0.55–0.98)
Specificity (95% CI)	0.67 (0.35–0.90)	0.64 (0.31–0.89)
ANCH‐B		
MCID improvement (95% CI)	**1.46** (0.10–2.80)	**2.38** (1.44–3.31)
MCID deterioration (95% CI)	**−3.21** (−4.90 to −1.53)	**−3.20** (−3.45 to −2.94)

*Note*: MCID improvement/deterioration: the difference between the mean change from HFMSE baseline score of the “stable” group and the mean score of the “improved” (or “deteriorated”) group. Bold indicates values of optimal cutoff.

Abbreviations: AUC, area under the curve; CI, confidence interval; MCID, minimal clinically important difference; SMA, spinal muscular atrophy.

Using the ANCH‐B approach, the MCID values for patient improvement were 1.5 and 2.4 for SMA type II and SMA type III patients, respectively. The estimated MCID for patient deterioration was −3.2 for both populations.

### Distribution‐based methods

HFMSE 1‐year change from baseline data was available for 321 subjects, 176 SMA II patients and 145 SMA III patients. Table 4 summarizes the baseline characteristics of the population analyzed.

**TABLE 4:** Characteristics at baseline of patients enrolled, by SMA type (whole population, distribution‐based).

Characteristic	All, *n* = 321	SMA type II, *n* = 176	SMA type III, *n* = 145
Observations per patient, median *n* (minimum–maximum)	2 (1–16)	2 (1–16)	2 (1–14)
Age, years, mean (SD)	12.36 (11.73)	10.34 (10.32)	14.82 (12.85)
Adults, *n* (%)	69 (21.50)	32 (18.18)	37 (25.52)
Sex, *n* (%)			
Female	114 (46.91)	78/174 (44.83)	36/69 (52.17)
Male	129 (53.09)	96/174 (55.17)	33/69 (47.83)
*SMN2* copy number, *n* (%)			
1	1 (0.31)	1 (0.57)	0 (0.00)
2	26 (8.10)	20 (11.36)	6 (4.14)
3	149 (46.42)	120 (68.18)	29 (20.00)
4+	23 (7.16)	1 (0.57)	22 (15.17)
Unknown	122 (38.01)	34 (19.32)	88 (60.69)
SMA function, *n* (%)			
Non sitter	48 (14.95)	46 (26.14)	2 (1.38)
Sitter	172 (53.58)	130 (73.86)	42 (28.97)
Walker	101 (31.46)	0 (0.00)	101 (69.66)

Abbreviations: SMA, spinal muscular atrophy.

The SEM in the whole type II SMA cohort was calculated to be 2.3, with subgroups subdivided based on age and functional status showing a range of 0.1–1.7. In the type III cohort, the SEM was found to be 3.4, ranging from 0.1 to 2.6. Regarding the EsCh, in the type II SMA cohort, it was determined to be 1.2, with a range of 0.5–1.8. In the type III cohort, the medium effect size change (EsCh05) was calculated to be 1.5, with a range of 0.6–2.1.

Table 5 shows details of the distribution‐based MDCs for HFMSE in SMA II and SMA III patients stratified by individual baseline characteristics.

**TABLE 5:** Minimal detectable change estimations for HFMSE for different distribution‐based methods applied to type II and III SMA patients, according to SMA type and function and HFMSE score, and substratified by age.

	N. oss	SEM	ESch
SMA II			
Whole population	574	2.3	1.2
Functional status and age			
Non sitters	110	0.2	0.5
≤5 yo	6	0.2	0.5
6–12 yo	37	0.2	0.5
13–20 yo	19	0.3	0.4
≥20 yo	48	0.1	0.4
Sitters	464	1.7	1.3
≤5 yo	168	1.7	1.4
6–12 yo	222	1.5	1.2
13–20 yo	58	0.5	0.5
≥20 yo	16	0.3	0.6
HFMSE score and age			
<10 HFMSE	177	0.4	1.0
≤5 yo	46	0.4	1.4
6–12 yo	66	0.4	0.9
13–20 yo	49	0.4	0.5
≥20 yo	16	0.3	0.6
10–22 HFMSE	219	0.8	1.2
≤5 yo	89	0.8	1.2
6–12 yo	121	0.8	1.2
13–20 yo	9	0.2	0.6
>22 HFMSE	68	0.9	1.7
≤5 yo	33	1.0	1.8
6–12 yo	35	0.8	1.6
SMA III			
Whole population	452	3.4	1.5
Functional status and age			
Non sitters	6	–^a^	–^a^
≥20 yo	6	–^a^	–^a^
Sitters	119	2.6	1.2
≤5 yo	3	0.1	0.6
5–8 yo	6	2.5	2.1
9–14 yo	49	2.4	2.1
15–20 yo	13	3.0	0.8
≥20 yo	48	2.0	1
Walkers	327	1.7	1.2
≤5 yo	46	1.5	1.1
5–8 yo	78	1.5	1.3
9–14 yo	125	1.6	1.7
15–20 yo	40	1.7	1.3
≥20 yo	38	1.8	1.2

Abbreviations: ESch, effect size change; HFMSE, Hammersmith Functional Motor Scale Expanded; N. oss, number of observations; SEM, standard error of measurement; SMA, spinal muscular atrophy; yo, years old.

^a^ All patients had an HFMSE score of 0; SEM and ESch were not computed.

Table S1 shows the distribution‐based values on the same cohort of patients included in the anchor‐based ones.

## DISCUSSION

Several natural history studies have shown that patterns of HFMSE changes can be very variable in type II and III SMA and that age and function have a significant impact on the changes [17, 29]. This has prompted a few questions on whether MCID or MDC values for HFMSE should be assessed taking these variables into account. This was also suggested by a recent study that, even if limited to adult patients and not reporting anchor‐based methods, showed that MCID varies in relation to SMA type and age [18].

One of the current challenges in calculating MCID in SMA is that this should be performed combining functional and patient‐reported data from untreated patients. As over the past few years most SMA patients have been treated with the available disease‐modifying therapies, this has strongly limited the possibility to perform new studies in untreated cohorts. The possibility to have access to a large international database with functional data in untreated patients and, even if limited to a smaller cohort, to concurrent patient/caregiver‐reported data, allowed us to establish MDC and MCID using anchor‐based methods in both type II and type III SMA cohorts.

Following suggestions from the FDA that MCID should be established through a combination of anchor‐based and distribution‐based methods, we used both approaches including different statistical methods [27]. First, we assessed possible differences between type II and III, and we found that there was always a difference in MCID values between the two cohorts, irrespective of the method used, confirming previous clinical observations of distinct patterns of progression.

Using the anchor‐method based on ROC curve analysis (ANCH‐A), a valuable tool for establishing cutoff points between improvement/stability and deterioration, we found that the optimal cutoff point was −2 for SMA type II patients and −4 for type III patients.

Using the anchor‐method based on standard error (ANCH‐B), where threshold values where determined in different categories to evaluate the significance of changes in patients' conditions, we identified that the MCID for improvement was 1.5 for SMA type II and 2.4 for type III. Meanwhile, the values for patient deterioration were −3.2 for both SMA types, aligning closely with the results obtained through ROC analysis.

In this paper, we were also interested in establishing possible differences within each cohort (type II and III) in relation to age and functional status. This was not possible for MCID because of the limited number of available concurrent questionnaires but could be assessed measuring MDC in the much larger cohort in whom HFMSE results were available without the restriction of having a concomitant questionnaire.

The results showed a large variability of MDC values among the individual age and functional subgroups within both the type II and type III cohorts. In type II the MDC value in the whole cohort was 2.3, but within the individual subgroups the values were always lower, with values as low as 0.1 in the nonsitter or adult subgroups. The maximum MDC value reached in the individual type II subgroups was 1.7, therefore much lower than the value of 2.3 found in the overall type II cohort. This discrepancy reflects the method of analysis, based on SDs. Whereas the individual age and functional subgroups were relatively homogeneous, the whole cohort, including all the type II patients from nonsitters to highly functioning sitters, is a much more heterogeneous cohort with subsequent larger standard variations. This discrepancy is even more obvious in the type III cohort, in whom the variability is larger because it also includes ambulant patients.

The implications of our findings are potentially of great significance for both clinicians and researchers, as they provide valuable insights into the variations in disease progression patterns among different subgroups of SMA patients. The incorporation of patient‐reported outcomes with ROC analysis (ANCH‐A) adds practicality and relevance to our study, making it applicable in real‐world clinical scenarios. Furthermore, the second method, based on standard error (ANCH‐B), adds robustness to our findings, as it aligns the MCID values with patient experiences and subjective assessments, providing a more holistic understanding of the clinical significance of changes observed in SMA patients.

In conclusion, despite the limitations of the small number of patients with completed questionnaires preventing us from obtaining MCID values in subgroups, as obtained for MDC, our results provide some reference data for both MCID and MDC that were quite concordant and complementary to each other. Our findings suggest that, when dealing with heterogeneous cohorts such as type II and type III SMA, the mean value of MCID in the whole cohort should be interpreted with caution and raise the issue of whether a single MCID value should be considered appropriate in heterogeneous diseases such as SMA, as also recently reported in other neuromuscular conditions, such as Duchenne muscular dystrophy, also showing variable progression of functional scores in relation to age and functional status [45].

These findings also raise the issue of whether, as in most countries the great majority of SMA patients are now under treatment and these have become standard of care, new MCID and MDC should be measured to reflect this "new natural history" in treated patients. This appears to be particularly relevant as, following the advent of the new therapies, both disease progression [14] and caregiver expectations [46] have significantly changed and are likely to have a strong impact on MCID.

## AUTHOR CONTRIBUTIONS

**Eugenio Mercuri:** Investigation; writing – original draft; writing – review and editing; methodology; supervision; conceptualization. **Giorgia Coratti:** Conceptualization; investigation; writing – original draft; methodology; writing – review and editing; data curation; supervision. **Francesca Bovis:** Conceptualization; methodology; formal analysis; writing – original draft; writing – review and editing. **Maria Carmela Pera:** Writing – original draft; writing – review and editing; validation; data curation. **Mariacristina Scoto:** Writing – original draft; writing – review and editing; data curation. **Jacqueline Montes:** Writing – original draft; writing – review and editing; data curation; investigation. **Amy Pasternak:** Investigation; writing – original draft; writing – review and editing; data curation. **Anna Mayhew:** Investigation; writing – original draft; writing – review and editing; data curation. **Robert Muni‐Lofra:** Investigation; writing – original draft; writing – review and editing; data curation. **Tina Duong:** Writing – original draft; writing – review and editing; data curation; investigation; methodology. **Annemarie Rohwer:** Investigation; writing – original draft; writing – review and editing; data curation. **Sally Dunaway Young:** Investigation; writing – original draft; writing – review and editing; data curation. **Matthew Civitello:** Investigation; writing – original draft; writing – review and editing; data curation. **Francesca Salmin:** Investigation; writing – original draft; writing – review and editing; data curation. **Irene Mizzoni:** Investigation; writing – original draft; writing – review and editing; data curation. **Simone Morando:** Investigation; writing – original draft; writing – review and editing; data curation. **Marika Pane:** Investigation; writing – original draft; writing – review and editing; supervision. **Emilio Albamonte:** Investigation; writing – original draft; writing – review and editing; supervision. **Adele D'Amico:** Investigation; writing – original draft; writing – review and editing; supervision. **Noemi Brolatti:** Investigation; writing – original draft; writing – review and editing; supervision. **Maria Sframeli:** Investigation; writing – original draft; writing – review and editing; supervision. **Chiara Marini‐Bettolo:** Investigation; writing – original draft; writing – review and editing; supervision. **Valeria Ada Sansone:** Investigation; writing – original draft; supervision. **Claudio Bruno:** Investigation; writing – original draft; writing – review and editing; supervision. **Sonia Messina:** Investigation; writing – original draft; writing – review and editing; supervision. **Enrico Bertini:** Investigation; writing – original draft; writing – review and editing; supervision. **Giovanni Baranello:** Investigation; writing – original draft; writing – review and editing; supervision. **John Day:** Investigation; writing – original draft; writing – review and editing; supervision. **Basil T. Darras:** Investigation; writing – original draft; writing – review and editing; supervision. **Darryl C. De Vivo:** Investigation; writing – original draft; writing – review and editing; supervision. **Michio Hirano:** Investigation; writing – review and editing; writing – original draft; supervision. **Francesco Muntoni:** Investigation; writing – original draft; writing – review and editing; methodology; supervision. **Richard Finkel:** Investigation; writing – original draft; writing – review and editing; methodology; supervision.

## CONFLICT OF INTEREST STATEMENT

G.C., M.C.P., M.Sc., J.M., A.P., A.M., R.M.L., T.D., S.D.Y., M.C., M.P., E.A., V.A.S., A.D., C.B., S.Me., E.B., G.B., and E.M. report personal fees from Biogen, Roche, Avexis, and Novartis outside the submitted work. G.C. reports personal fees from Genesis Pharma and Biologix outside the submitted work. None of the other authors has any conflict of interest to disclose.

## Supporting information


**TABLE S1** Minimal detectable change estimations for Hammersmith Functional Motor Scale Expanded for different distribution‐based methods applied to type II and III spinal muscular atrophy (SMA) patients, according to SMA type in the subgroup of patients (*n* = 76) with patient‐reported clinical perception questionnaire available.

## Data Availability

The data that support the findings of this study are available from the corresponding author upon reasonable request.
