# Supplementary material for: Determining minimal clinically important differences in the Hammersmith Functional Motor Scale Expanded for untreated spinal muscular atrophy patients: An international study
Source: Eur J Neurol. 2024 Apr 24;31(8):e16309. doi: 10.1111/ene.16309 (PMC11236020; doi:10.1111/ene.16309)
Supplement: Supplementary file 1 — TABLE S1 Minimal detectable change estimations for Hammersmith Functional Motor Scale Expanded for different distribution‐based methods applied to type II and III spinal muscular atrophy (SMA) patients, according to SMA type in the subgroup of patients (n = 76) with patient‐reported clinical perception questionnaire available. [file ENE-31-e16309-s001.docx]

**Supplementary table 1. MDC estimations for HFMSE for different distribution-based method applied to type II and III SMA patients, according to SMA type in the subgroup of patients (N=76) with patient-reported clinical perception questionnaire available.** Key to table: SEM= standard error of measurement; ESch: effect size change; SDbas=standard deviation at baseline; SDch= standard deviation of the difference between the evaluation at baseline and at 12-month.

|  |  | **SMA type II** | **SMA type III** |
| --- | --- | --- | --- |
| **SEM** | SDbas*****$\sqrt{1-0.959}$ | 1.9 | 2.4 |
| **ESch** | 0.5*SDch | 1.6 | 2.3 |
